# Supplementary material for: Computational exploration of the global microbiome for antibiotic discovery
Source: bioRxiv. 2023 Sep 11:2023.08.31.555663. Originally published 2023 Aug 31. Preprint. [Version 2] doi: 10.1101/2023.08.31.555663 (PMC10491242; doi:10.1101/2023.08.31.555663)
Supplement: Supplement 10 [file NIHPP2023.08.31.555663v2-supplement-10.pdf]

bioRxiv preprint doi: https://doi.org/10.1101/2023.08.31.555663; this version posted September 11, 2023. The copyright holder for this preprint (which was not certified by peer review) is the author/funder, who has granted bioRxiv a license to display the preprint in perpetuity. It is made available under aCC-BY 4.0 International license.

Supplemental Information

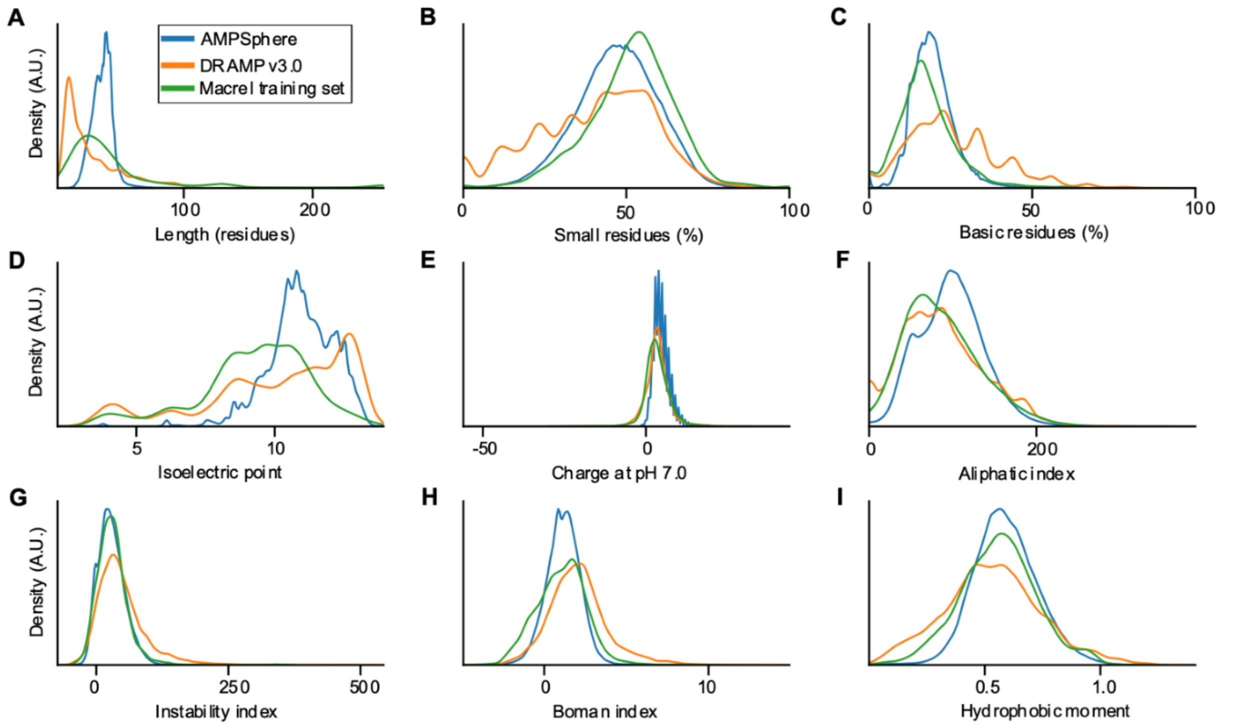

Figure SI1. General physical-chemical features of c_AMPs in AMPSphere and validated databases of antimicrobial peptides. All graphs are drawn using the continuous probability density curve and given as functions of the arbitrary density units. The c_AMPs from AMPSphere retain the same predicted biochemical properties of those from validated AMPs, although with an average length longer than the peptides in the training set of Macrel [S1] and DRAMP 3.0 [S2]. (***A***) The distribution of peptide length in residues across the AMPSphere. (***B***) Distribution of proportions of residues with small side chains [A, B, C, D, G, N, P, S, T, V] per AMP. (***C***) Distribution of proportions of basic residues [H, R, K] per AMP. (***D***) Distribution of isoelectric points in AMPSphere peptides. (***E***) Distribution of peptide charges at pH 7.0. (***F***) Distribution of aliphatic index in peptides from AMPSphere. (***G***) Distribution of instability index in AMPSphere. (***H***) Distribution of Boman index in AMPSphere. (***I***) Distribution of hydrophobic moments in AMPSphere.

bioRxiv preprint doi: https://doi.org/10.1101/2023.08.31.555663; this version posted September 11, 2023. The copyright holder for this preprint (which was not certified by peer review) is the author/funder, who has granted bioRxiv a license to display the preprint in perpetuity. It is made available under aCC-BY 4.0 International license.

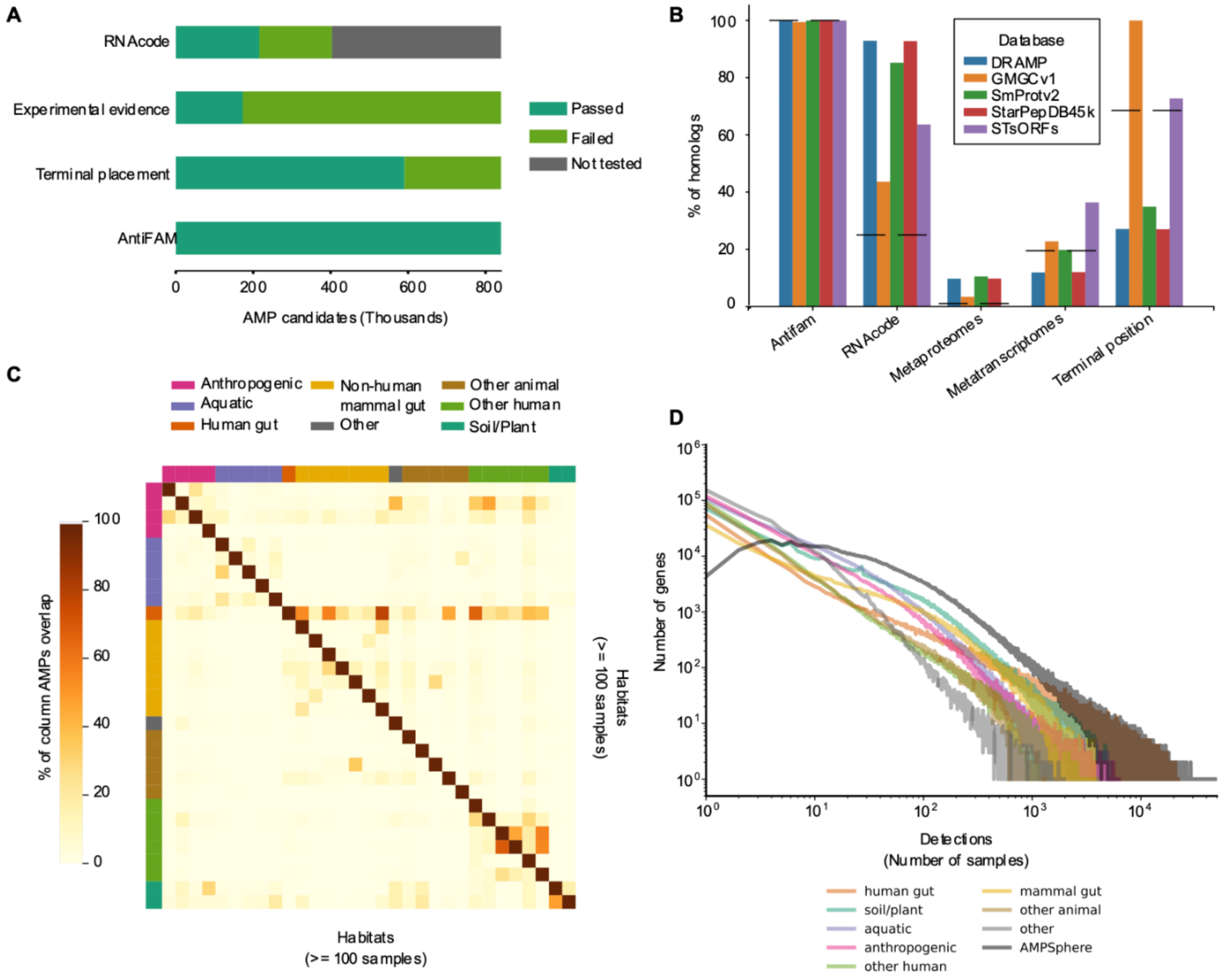

Figure SI2. c_AMPs quality and habitat distribution. (***A***) Quality assessment of AMPSphere reveals most of the peptides passing at least 1 of the tests*.* The RNAcode test depends on gene diversity, which is very low for AMPSphere, and therefore, determines a low rate of positives among our candidates*.* (***B***) c_AMPs homologous to databases of validated bioactive peptides also showed a higher average quality of these datasets. (***C***) The limited overlap of c_AMPs among habitats argues in favor of using habitat groups to gain resolution. Note that the group of habitats with the highest paired overlaps belong to human body sites and samples from human guts and non-human mammalian guts. Only habitats with at least 100 samples were shown. (***D***) It is also possible to observe the great proportion of rare genes in AMPSphere from different habitat groups, in which few genes are largely detected.

bioRxiv preprint doi: https://doi.org/10.1101/2023.08.31.555663; this version posted September 11, 2023. The copyright holder for this preprint (which was not certified by peer review) is the author/funder, who has granted bioRxiv a license to display the preprint in perpetuity. It is made available under aCC-BY 4.0 International license.

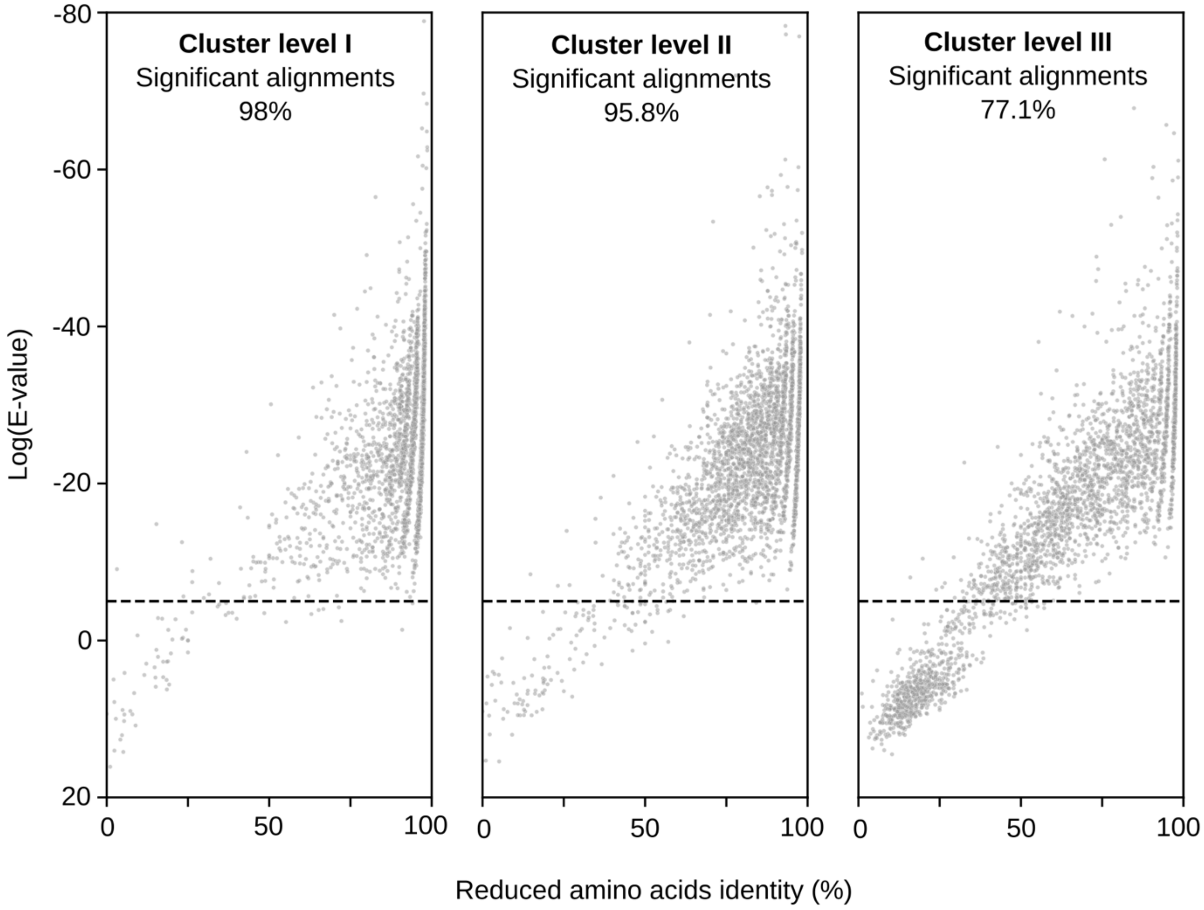

Figure SI3. Clustering validation of families. To validate the clustering procedure using a reduced amino acid alphabet, samples of 1,000 peptides were randomly drawn from AMPSphere (excluding representative sequences) and aligned against their cluster representatives. Three different levels (I, II, and III) of clustering were tested. The E-values were computed per alignment and plotted against the corresponding alignment identity. The averaged proportion of significant alignments is shown above each graph.

bioRxiv preprint doi: https://doi.org/10.1101/2023.08.31.555663; this version posted September 11, 2023. The copyright holder for this preprint (which was not certified by peer review) is the author/funder, who has granted bioRxiv a license to display the preprint in perpetuity. It is made available under aCC-BY 4.0 International license.

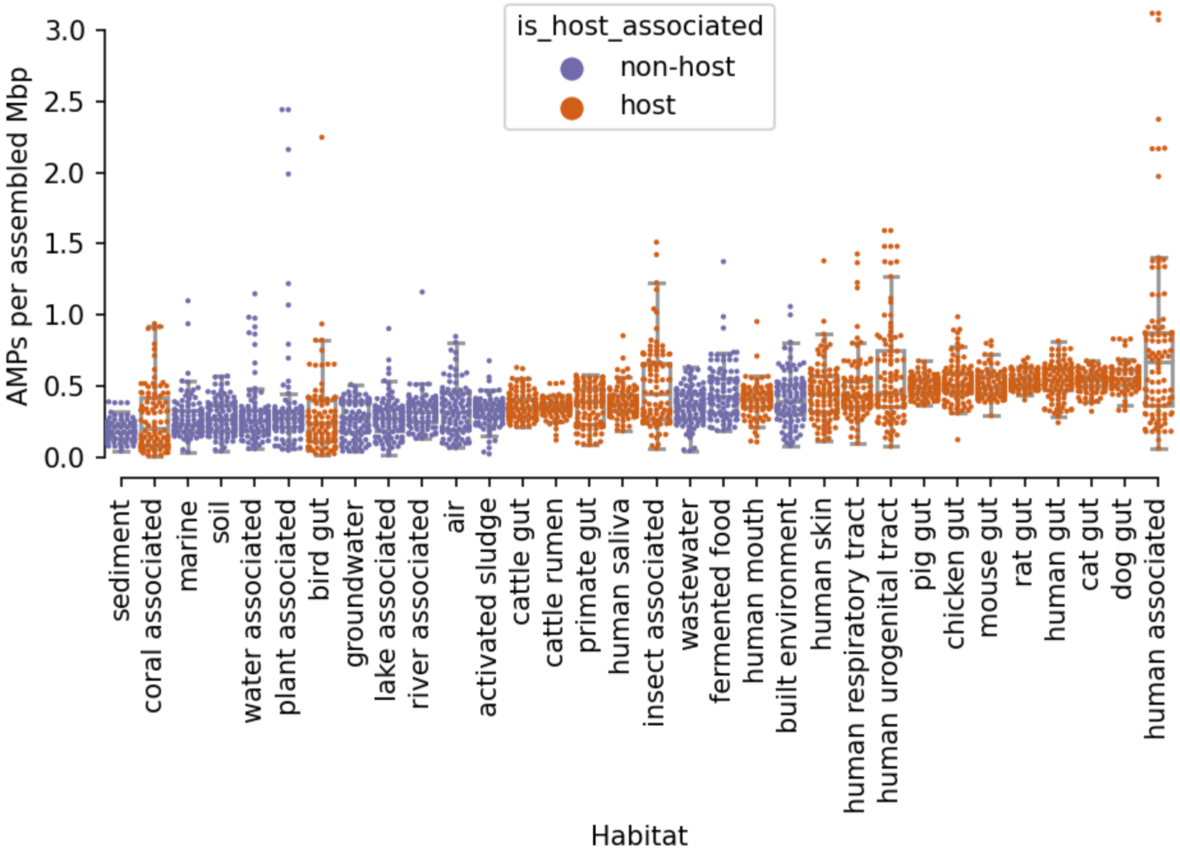

Figure SI4. Host-associated habitats are denser in c_AMPs. The c_AMP density (given as c_AMP genes per assembled Mbp) was computed per sample and plotted per habitat. Only habitats with at least 100 samples were used. To favor a good visualization, we sampled 2,000 dots to plot their distribution trends. habitats were colored by their ontology, orange for the animal host-associated samples and purple for the non-animal-host-associated samples. Host-associated habitats cluster at the right portion of the graph with some anthropogenic habitats, which are closer to animal host-associated samples than habitat samples. Few exceptions, such as coral-associated and bird gut habitats cluster with non-animal-host associated habitats.

bioRxiv preprint doi: https://doi.org/10.1101/2023.08.31.555663; this version posted September 11, 2023. The copyright holder for this preprint (which was not certified by peer review) is the author/funder, who has granted bioRxiv a license to display the preprint in perpetuity. It is made available under aCC-BY 4.0 International license.

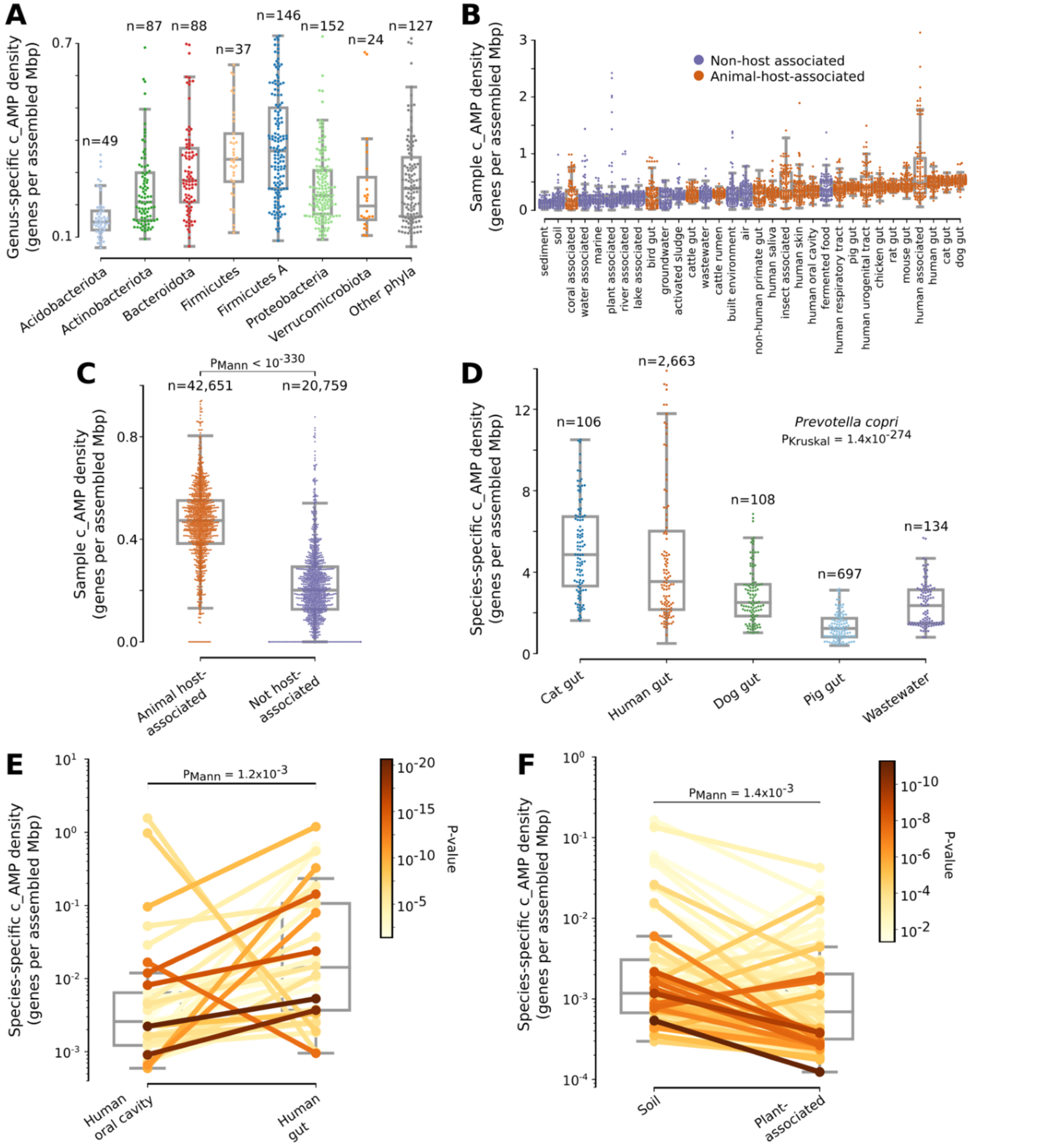

Figure SI5. Quality-controlled c_AMPs do not change the differences observed for c_AMP density across several bases of comparison. To evaluate the effects of possible gene fragments, we filtered off genes not having a stop codon upstream of them and recalculated the c_AMP density (given as c_AMP genes per assembled Mbp). The same results could be observed in the correspondent analysis using all c_AMPs. ***(A)*** The main phyla contributing with more c_AMPs in AMPSphere and the distribution of these densities in each of those groups by genera - correspondent

bioRxiv preprint doi: https://doi.org/10.1101/2023.08.31.555663; this version posted September 11, 2023. The copyright holder for this preprint (which was not certified by peer review) is the author/funder, who has granted bioRxiv a license to display the preprint in perpetuity. It is made available under aCC-BY 4.0 International license.

to ***Fig. 4C***. ***(B)*** Animal host-associated habitats are denser in c_AMPs - correspondent to ***Fig. SI4. (C)*** Animal host-associated habitats have a higher sample c_AMP density when compared to those non-host-associated - correspondent to ***Fig. 5A***. ***(D)*** Hosts are a factor for variation of c_AMP density in *Prevotella copri*, presenting a higher ρAMP in cat and human guts compared to the same species in guts of pigs and dogs - correspondent to ***Fig. 5B***. 106 randomly selected points are shown for each host. ***(E)*** Species-specific ρ_{AMP} of microbes from the human gut are higher, when compared against the same species found in the human oral cavity - correspondent to ***Fig. 5C***. ***(F)*** For non-animal hosts, the species-specific ρ_{AMP} of microbes from the soil is higher when compared against the same species found in plant-associated samples - correspondent to ***Fig. 5D***. For panels ***E*** and ***F*** the significance was color-encoded using a $Log_{10}(P_{Mann})$ scale.

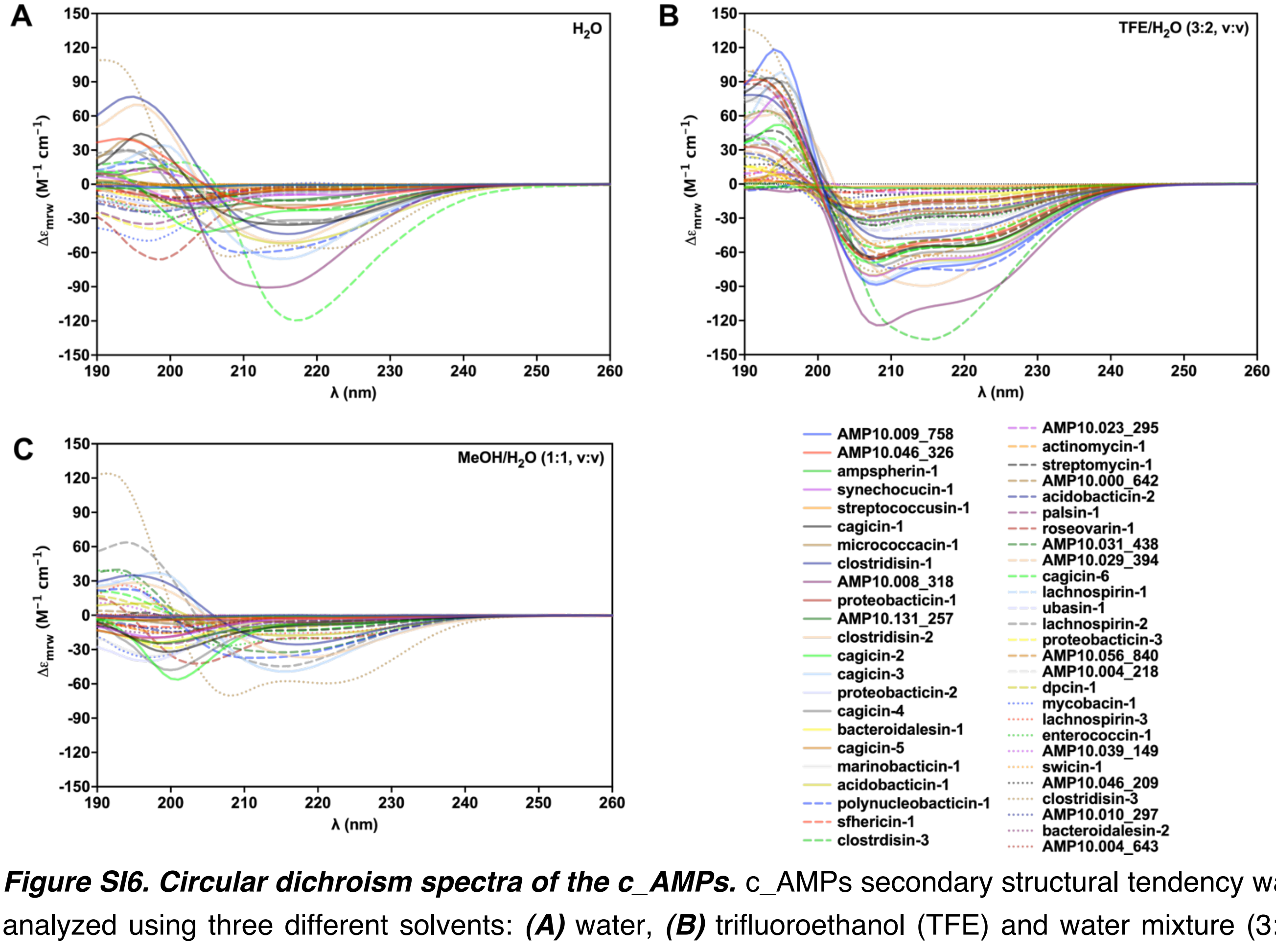

Figure SI6. Circular dichroism spectra of the c_AMPs. c_AMPs secondary structural tendency was analyzed using three different solvents: ***(A)*** water, ***(B)*** trifluoroethanol (TFE) and water mixture (3:2, V:V), and ***(C)*** methanol (MeOH) and water mixture (1:1, V:V). The experiments were carried out at 25

bioRxiv preprint doi: https://doi.org/10.1101/2023.08.31.555663; this version posted September 11, 2023. The copyright holder for this preprint (which was not certified by peer review) is the author/funder, who has granted bioRxiv a license to display the preprint in perpetuity. It is made available under aCC-BY 4.0 International license.

°C, and the circular dichroism spectra shown are an average of three accumulations obtained using a quartz cuvette with an optical path length of 1.0 mm, ranging from 260 to 190 nm at a rate of 50 nm·min^{-1} and a bandwidth of 0.5 nm. All peptides were tested at a concentration of 50 µmol·L^{-1}, with respective baselines recorded prior to measurement. A Fourier transform filter was applied to minimize background effects.

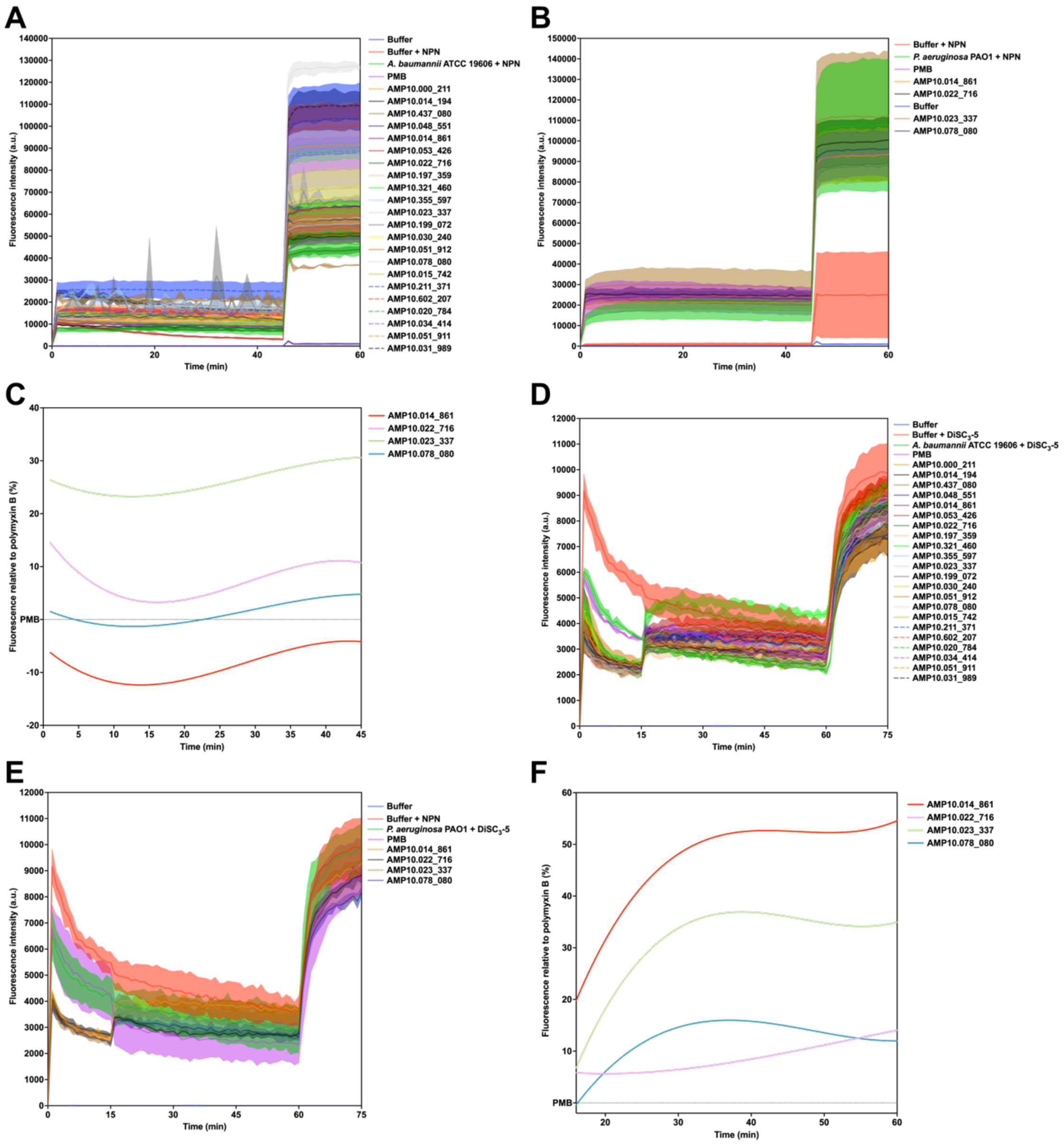

Figure SI7. Mechanism of action of AMPSphere peptides. Permeabilization assays with the fluorescent probe 1-(N-phenylamino)naphthalene (NPN) showing the effect of AMPSphere peptides on ***(A)*** *A. baumannii* ATCC 19606 and ***(B)*** *P. aeruginosa* PA01 cells, and ***(C)*** Fluorescence values relative to polymyxin B (PMB, positive control) of the fluorescent probe 1-(N-phenylamino)naphthalene (NPN) that indicate outer membrane permeabilization of *P. aeruginosa* PA01 cells. Depolarization assays with the hydrophobic probe 3,3′-dipropylthiadicarbocyanine iodide [DiSC3-(5)], effect of AMPSphere peptides in ***(D)*** *A. baumannii* ATCC 19606 and ***(E)*** *P. aeruginosa* PA01 cells. ***(F)***

bioRxiv preprint doi: https://doi.org/10.1101/2023.08.31.555663; this version posted September 11, 2023. The copyright holder for this preprint (which was not certified by peer review) is the author/funder, who has granted bioRxiv a license to display the preprint in perpetuity. It is made available under aCC-BY 4.0 International license.

Fluorescence values relative to PMB (positive control) of 3,3′-dipropylthiadicarbocyanine iodide [DiSC3-(5)], a hydrophobic fluorescent probe, used to indicate cytoplasmic membrane depolarization of *P. aeruginosa* PA01 cells. Data in ***(A)***, ***(B)***, ***(D)***, and ***(E)*** are the mean plus and minus the standard deviation.

Excel Tables

Table SI1. Metadata and description of (meta)genomes used in AMPSphere. The sample is identified by its access code in ENA, the habitat shows the type of habitat this sample was retrieved from. Other data about the sequencing, such as the number of raw inserts and the number of assembled base pairs (bp) are also available along with the information on N50. The number of predicted complete large ORFs (>100 amino acids) and smORFs (10-100 amino acids) is shown (ORFs+smORFs) along with the number of smORFs alone and the predicted non-redundant c_AMPs.

Table SI2. c_AMP distribution in the habitat groups. The habitats grouped under each class are shown along with the number of genes encoding the non-redundant c_AMPs, the number of c_AMP clusters in total, and the number of clusters containing ≥8 c_AMPs (c_AMP families).

Table SI3. Ortholog groups (OGs) enrichment in the hits to the GMGCv1 [S3]***.*** Top hits were assessed and the proportion of OGs from eggNOG 5 [S4] was compared using the number of c_AMPs affiliating to homologs of a given OG and the total number of OGs found in the homologs of c_AMPs (156,711) in the comparison to the GMGCv1 [S3]. As a background measure, we used the counts of a given OG in the redundant set of genes belonging to GMGCv1 [S3] and the total number of OGs found in the redundant GMGC catalog [S3] (9,180,087,363). Enrichment in the c_AMPs set was given as the fold-change calculated for each given OG in relation to that expected in the GMGCv1 [S3]. P-values were adjusted using Holm-Sidak and only significant hits ($P < 0.05$) were shown.

Table SI4. c_AMP genome context in comparison to families with proteins of different sizes. The proportion of families of proteins of different sizes (all lengths and only ≤ 50 amino acids) is presented in comparison to the proportion of mapped AMPs (55,191) with genome contexts involving a given Kyoto Encyclopedia of Genes and Genomes – KEGG ortholog pathway [S5] shown with their accession code and description.

Table SI5. Permutations with random families of different sizes show that the genome context of c_AMPs is different from other protein families. We performed 10,000 random samplings of 55,191 protein families of all lengths and only smaller than 50 amino acids. These families were assessed regarding their genome neighborhood conservation. It was found that the values found in AMPSphere are mostly different from those observed for any other protein families across genome contexts involving known functions, antibiotics synthesis/resistance, and antibiotic resistance genes from CARD [S6].

bioRxiv preprint doi: https://doi.org/10.1101/2023.08.31.555663; this version posted September 11, 2023. The copyright holder for this preprint (which was not certified by peer review) is the author/funder, who has granted bioRxiv a license to display the preprint in perpetuity. It is made available under aCC-BY 4.0 International license.

Table SI6. AMPs with conserved genome contexts sharing KEGG ortholog groups (KO) with gene neighbors. AMPs were annotated with eggNOG mapper [S7] and those harboring KOs were included in this analysis. It is shown for each KO a brief description of its activity, the total number of AMPs annotated, the number of those with conserved neighbors (conservation score > 0.9), and the number of AMPs assigned to a given KO inserted in a conserved neighborhood containing other genes annotated to that KO (the intersection before the 2 previous columns).

Table SI7. c_AMP density across different genera. The number of redundant c_AMP genes as well as their respective assembled base pairs is presented with the calculated AMP density. The error on the AMP density measure is also shown evidence that most of the genera present an error above 10% (our cutoff).

Table SI8. Differential species-specific c_AMP density across habitats. The tested species had their densities (as c_AMP genes per assembled gigabase pairs) compared across samples from different habitats (Habitats A and B), and the number of samples for each habitat was also registered after eliminating the outliers using Tukey's fences with k = 1.5 (# Samples A and B). The average c_AMP density for each species in each habitat was registered along with its standard deviation (Avg. c_AMP density and Std. c_AMP density). The Mann-Whitney U test was applied to each pair of habitats and later corrected using the Holm-Sidak method, shown as the 'Adjusted P-value'. Comparisons considered species present in at least 10 samples for each habitat with ≥ 100 samples presenting c_AMPs. Only significant comparisons were shown.

Table SI9. Metatranscriptomes and metaproteomes used in the verification for experimental signals of transcription and/or translation of c_AMP genes from AMPSphere. Metatranscriptomes from EMBL-ENA were used in the comparisons with genes in AMPSphere to verify signals of transcription in datasets *ad hoc*. The datasets from the Proteomics Identification Database (PRIDE) - EMBL-EBI were also used in the comparison to c_AMPs and verified the same peptides in datasets *ad hoc*.

Supplemental references

S1. Santos-Júnior CD, Pan S, Zhao X-M, Coelho LP. Macrel: antimicrobial peptide screening in genomes and metagenomes. *PeerJ* 2020; ***8***:e10555.

S2. Shi G, Kang X, Dong F, *et al.* DRAMP 3.0: an enhanced comprehensive data repository of antimicrobial peptides. *Nucleic Acids Research* 2021.

S3. Coelho LP, Alves R, del Río ÁR, *et al.* Towards the biogeography of prokaryotic genes. *Nature* 2022; ***601***:252–256.

S4. Huerta-Cepas J, Szklarczyk D, Heller D, *et al.* eggNOG 5.0: a hierarchical, functionally and phylogenetically annotated orthology resource based on 5090 organisms and 2502 viruses. *Nucleic Acids Research* 2019; ***47***:D309–D314.

bioRxiv preprint doi: https://doi.org/10.1101/2023.08.31.555663; this version posted September 11, 2023. The copyright holder for this preprint (which was not certified by peer review) is the author/funder, who has granted bioRxiv a license to display the preprint in perpetuity. It is made available under aCC-BY 4.0 International license.

S5. Kanehisa M, Sato Y. KEGG Mapper for inferring cellular functions from protein sequences. *Protein Sci* 2020; ***29***:28–35.

S6. Alcock BP, Raphenya AR, Lau TTY, *et al.* CARD 2020: antibiotic resistome surveillance with the comprehensive antibiotic resistance database. *Nucleic Acids Res* 2020; ***48***:D517–D525.

S7. Cantalapiedra CP, Hernández-Plaza A, Letunic I, Bork P, Huerta-Cepas J. *eggNOG-mapper v2: Functional Annotation, Orthology Assignments, and Domain Prediction at the Metagenomic Scale*.; 2021:2021.06.03.446934.

·
